# Moving primary prevention into a tertiary care hospital?

**DOI:** 10.1265/ehpm.25-00263

**Published:** 2025-09-23

**Authors:** Diane Geindreau, Lou Martineau, Aymery Constant, MECC Angers Group, Alexis Descatha, Celine Schnebelen

**Affiliations:** 1 University Hospital (CHU Angers), Federation of Prevention and Health Promotion, Angers, France; 2 CNAM, National Conservatory of Arts and Crafts, Paris, France; 3 Univ Angers, CHU Angers, Univ Rennes, Inserm, EHESP, Irset (Institut de recherche en santé, environnement et travail) - UMR_S 1085, IRSET-ESTER, SFR ICAT, CAPTV/ Prevention, Angers, France; 4 Indiana University School of Public Health-Bloomington (IU SPH-B), IN, USA; 5 Department of Occupational Medicine, Epidemiology and Prevention, Donald and Barbara Zucker School of Medicine, Hofstra/Northwell, USA

**Keywords:** Public health, Prevention, MECC, Making every contact count, Occupational, Addiction, Smoking, Intervention

Dear Editors,

The importance of prevention is now well established around the world, sometimes even threatened by new regulations [1]. However, the separation between treatment and prevention is still prevalent in most Western cultures. For instance, application in prevention of life course epidemiology is still rare [2]. Although major guidelines emphasize the importance of primary prevention, they often refer only to primary care, and hospital settings are rarely considered as sites of prevention: outside of Ireland and the United Kingdom, there is no real on primary prevention in hospitals in Europe, which may be seen as a missed opportunity [3]. In a global context where prevention is the poor relation of clinical medicine, we argue that integrating primary prevention into the care of patients with other diseases may be an effective strategy to improve prevention. We report here on our experience with integrated primary prevention in a tertiary care – University hospital.

After review of evidence of what is effective and what is feasible in terms of resources in our university hospital - more than 200,000 patients every year, more than 7,000 workers, and the referent state hospital - a collaborative team developed a long-term strategic roadmap for the implementation of prevention and health promotion policies (See Fig. 1).

**Fig. 1:**
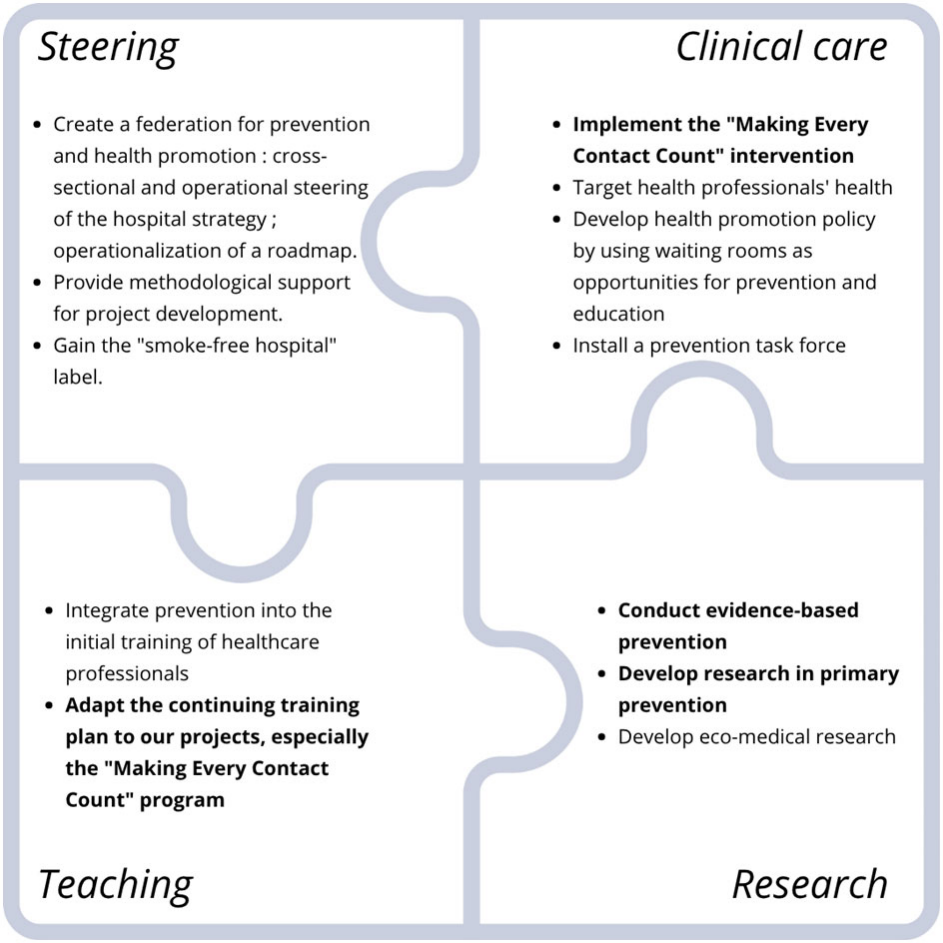
Institutional Roadmap – Prevention and Public Health 2024–2034. The sections that are in bold font pertain to the execution of the Making Every Contact Counts program.

This roadmap is divided into four strategic areas: steering, clinical care, teaching, and research. The “steering axis” involves creating a collaborative, multidisciplinary federation to pilot the roadmap and provide methodological support for project development. The “clinical care” axis is related to implementing prevention programs within care practices for patients and professionals. The “teaching” axis relates to integrating prevention and health promotion into initial and continuous training for healthcare professionals. Finally, the “research” axis aims to target evidence-based interventions for our policy development and transfer these interventions into our specific context to contribute to the scientific literature on this topic.

This letter describes a pilot study that ties into this roadmap through the clinical care, teaching, and research axes: the implementation of the “Making Every Contact Count” program in a University hospital. The aim of the pilot study is to assess the feasibility of implementing the MECC program in two voluntary units of our hospital without additional funding, before implementing it across the whole hospital.

Smoking and excessive alcohol consumption were selected as the initial risk factors for the pilot study because of their impact on the global burden of disease [4]. Making Every Contact Count (MECC), a person-centered approach to promoting health behavior change, was implemented in two different settings that volunteered to participate in the experiment: an emergency department and a surgical outpatient clinic from our University hospital [5, 6]. A core team included a physician, administrative director, project manager and a researcher, while an extended committee included the core team and other practitioners (MD, RN…) as well as hospital administrators, students, and patients. The MECC is an approach based on behavior change theory and techniques, such as screening, brief intervention, and referral to treatment (SBIRT), as well as healthy conversation skills [7, 8]. To develop a clear methodology for implementing and teaching these techniques, we worked closely with a professor of cognitive and behavioral sciences.

This pilot study includes different stages of intervention implementation. In a first place, the core team developed with the IT department a conversational tool for healthcare professionals to administer the MECC. This tool is a questionnaire that prompts additional questions when patients answer “yes” to certain ones. It is divided into three sections following the MECC principles: screening, intervention, and guidance toward specialized services (see Table 1). It can be used as both a conversational guide and a data collection tool, with the data integrated into the electronic medical record. Whenever a patient meets with a healthcare professional, the professional will use the guide to ask the patient about his or her smoking and drinking habits. A “yes” answer will prompt further questions. With the patient’s consent, the professional can initiate a brief intervention. The patient will be asked about his or her motivation to quit, and information about the benefits of quitting/adopting new habits will be provided. Nicotine replacement therapy (NRT) may be prescribed. To conclude the intervention, the professional refers the patient to specialists and provide more information on ways to receive support and build on this first step toward behavior change. The professional also has the option of indicating that he or she did not have time to proceed with the brief intervention due to unit organization or patient flow. All data are collected and analyzed on a monthly basis for monitoring and research purposes.

**Table 1:** Summary of the conversational tool specially designed for the MECC (Making Every Contact Count) implementation in our university hospital.

***Screening section for alcohol***	***Intervention section*** ***(Prompted if “yes” to screening questions.)***	***Orientation section***
• **Do you ever drink alcohol?** • Number of units per occasion • Frequency	**The patient’s consent is necessary, and if a risk factor is identified:** • Assessment of the motivation to undergo change • Assessment of the perceived capability to undergo change • Delivery of some educational materials • Prescription of Nicotine replacement therapy	**Orientation towards:** • Specialists in inpatient or outpatient settings • General practitioner • Prescription of Nicotine replacement therapy
***Screening section for tobacco smoking***		
• **Do you ever smoke tobacco?** • Quantity • Tobacco type		
***Screening section for marijuana smoking***		
• **Do you ever smoke marijuana?** • Quantity		

In a second place, the core team developed specialized training supervised by our team and addiction specialists/psychiatrists. This two-day training is designed for nurses and nurse assistants from the two voluntary care units. Three sessions are planned each year, with space for 12 professionals per session. To date, one session have been delivered, training 10 professionals (7 nurses and 3 nurse assistants). Day one covers the theoretical aspects of MECC principles, behavioral change theories and techniques, and alcohol and smoking epidemiology. Day two focuses more on practical application, simulation, and tool usage (see Supplementary Materials I). The objectives are to enable professionals to develop robust theoretical and practical skills, practice using the tools designed for MECC purposes, meet all the project partners, and raise any questions or concerns about their daily practices and how MECC delivery can fit in.

In parallel, a health promotion program was also proposed to all workers at our tertiary care hospital. Indeed, the literature on the implementation of the MECC program shows how it may affect the habits of both patients and professionals [8]. Therefore, we aim to provide them with worksite health-promoting activities that align with this preventive shift [9].

Between February (right after the first training session) and June 2025, 10 trained healthcare professionals delivered a MECC intervention to 124 patients (average age of 44 years) from their units. Outcomes were (based on conversational tool): total number of screenings and number of positive screening, type of addiction, number of interventions, and number of orientations, age and gender. The proof of concept was effective; 59 patients had at least one addiction, and 45.7% (27 patients) received a brief intervention. Systemizing discussions with patients across the health care system and strengthening the core competencies of caregivers to support patients in behavior change is a valuable response. This pilot study, successfully developed based on the human resources involved, illustrates the feasibility and benefits of conducting prevention across the health care system. If we are to actively promote prevention for our patients, we shall integrate it into the treatment of all users throughout the healthcare system. A key strength lies in the promotion of health for both healthcare workers and patients.

This pilot study illustrates that the MECC methodology, when properly implemented and delivered, is capable of integrating prevention at the heart of the hospital environment and our approach to patient care, thereby addressing some of the most pressing challenges facing our healthcare systems, though time and resources will be needed.

## Supplementary information

**Additional file 1:** **SUPPLEMENTARY MATERIAL** I. MECC Training guide based on theoretical content and practice.

## Declarations

### Availability of data and materials

Not relevant.
