# Supplementary material for: Moving primary prevention into a tertiary care hospital?
Source: Environ Health Prev Med. 2025 Sep 23;30:76. doi: 10.1265/ehpm.25-00263 (PMC12483757; doi:10.1265/ehpm.25-00263)
Supplement: Supplementary file 1 — Additional file 1: SUPPLEMENTARY MATERIAL I. MECC Training guide based on theoretical content and practice. [file ehpm-30-076-s001.DOCX]

SUPPLEMENTARY MATERIAL

I. MECC Training guide based on theoretical content and practice

| **Theoretical contents** |
| --- |
| - Role of healthcare facilities in primary prevention - Definition of the MECC - Position of the MECC within the institutional roadmap |
| - Epidemiology of certain diseases - Addiction and risk factors |
| - The influence of behaviors on health - Com-B model |
| - Screening, Brief Intervention and Referral to Treatment (SBIRT) |
| - Specific features of care for patients exposed to tobacco and alcohol risks - Epidemiology of smoking in France - Identify, advise and offer help to smokers - Substitute and how they are covered in France |

| **Practice** |
| --- |
| - Care pathways for tobacco and alcohol - Introduction of different local partners |
| - Introduction to the conversational tool - Practical application with tobacco and marijuana cases - Practical application with alcohol cases |
